# Effects of Sewage Discharge on Trophic State and Water Quality in a Coastal Ecosystem of the Gulf of California

**DOI:** 10.1155/2014/618054

**Published:** 2014-02-24

**Authors:** Héctor Hugo Vargas-González, José Alfredo Arreola-Lizárraga, Renato Arturo Mendoza-Salgado, Lía Celina Méndez-Rodríguez, Carlos Hernando Lechuga-Deveze, Gustavo Padilla-Arredondo, Miguel Cordoba-Matson

**Affiliations:** ^1^Centro de Investigaciones Biológicas del Noroeste, S. C. (CIBNOR, S.C.), 85454 Guaymas, SON, Mexico; ^2^Centro de Investigaciones Biológicas del Noroeste, S. C. (CIBNOR, S.C.), 23090 La Paz, BCS, Mexico

## Abstract

This paper provides evidence of the effects of urban wastewater discharges on the trophic state and environmental quality of a coastal water body in a semiarid subtropical region in the Gulf of California. The concentrations of dissolved inorganic nutrients and organic matter from urban wastewater primary treatment were estimated. La Salada Cove was the receiving water body and parameters measured during an annual cycle were temperature, salinity, dissolved oxygen, nitrite, nitrate, ammonia, orthophosphate, and chlorophyll *a*. The effects of sewage inputs were determined by using Trophic State Index (TRIX) and the Arid Zone Coastal Water Quality Index (AZCI). It was observed that urban wastewater of the city of Guaymas provided 1,237 ton N yr^−1^ and 811 ton P yr^−1^ and TRIX indicated that the receiving water body showed symptoms of eutrophication from an oligotrophic state to a mesotrophic state; AZCI also indicated that the environmental quality of the water body was poor. The effects of urban wastewater supply with insufficient treatment resulted in symptoms of eutrophication and loss of ecological functions and services of the coastal ecosystem in La Salada Cove.

## 1. Introduction

Mexico has 110 million inhabitants, of which ~15% live in the coastal zone [1] in 156 coastal municipalities located along ~11,000 km of coastline [2], and one of the main environmental problems on the national level is that most coastal cities are characterized by the insufficient treatment of municipal wastewater [3]. It is estimated that 58% of the wastewater from urban centers and 81% of industrial wastes are discharged directly into water bodies with no or inadequate treatment resulting in ~73% of the water bodies being contaminated [4]. Industrial or municipal sewage is fast emerging as an environmental problem whereby untreated or poorly treated water is discharged directly into coastal water bodies making them highly susceptible to eutrophication.

In general, it has been observed that tropical coastal ecosystems of developing countries have limited treatment processes of wastewater, causing possibly eutrophication problems of adjacent coastal systems due to nutrient inputs from anthropogenic sources, as well as the increased intensity and duration of solar radiation [5, 6].

The current state of knowledge of coastal eutrophication paradigm is changing and evolving from the traditional limited definition: an increase in the rate of supply of organic matter to an ecosystem [7]. Coastal eutrophication should now also include a macroscopic component where it takes into account the impacts of the drivers of global change and the increases in world population on the coasts with up to 50% of the earth's surface having been converted to agricultural and livestock production [8]. For this reason, it is imperative to increase the studies providing evidence of change in coastal ecosystems in order to evaluate the responses to nutrient stimulation [9].

Eutrophication is a process that involves an increase in the trophic status of a water body [7] and therefore the understanding of changes in the trophic status has been an important issue that has motivated the design of indexes and models. An example of this is the TRIX index (Trrophic Index) [10] and AZCI (Arid Zone Coastal Water Quality Index) [11]. The TRIX has been applied mainly in-coastal water bodies of Europe [12–17]. The AZCI has been applied in coastal water bodies in arid regions of Northwest Mexico [11, 18].

This study was conducted at La Salada Cove, a coastal subtropical water body in an arid region that receives urban sewage from the city of Guaymas (~120,000 inhab.) located on the east coast of the Gulf of California. The objective is to determine the effects of nutrient inputs on trophic state and environmental quality of the ecosystem.

## 2. Materials and Methods

### 2.1. Study Area

**Figure 1:**
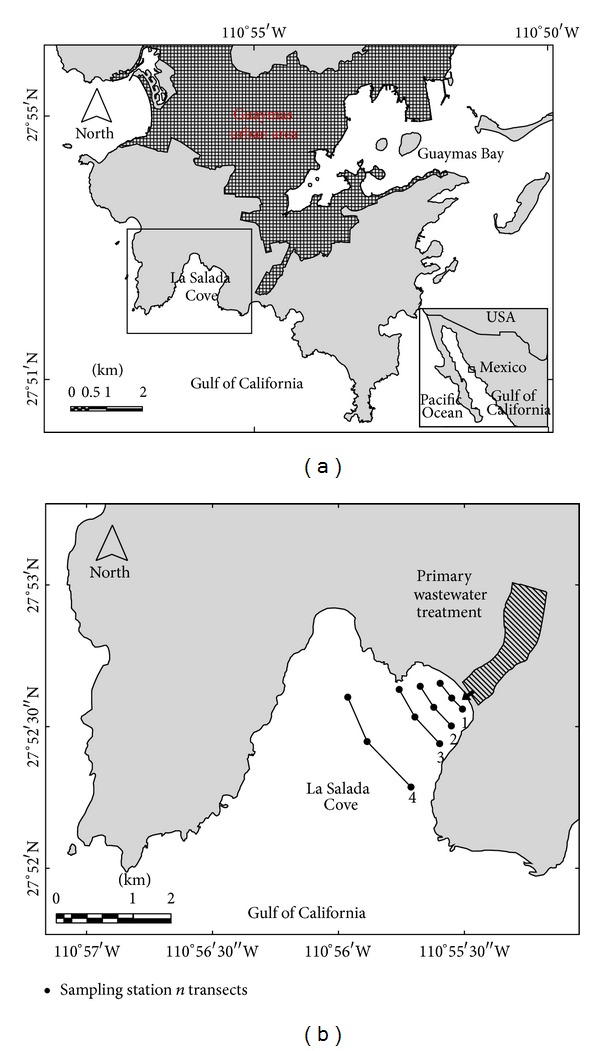
La Salada Cove, including urban area from Guaymas city and showing sampling stations and transects.

La Salada Cove is located on the Gulf of California in the State of Sonora, Mexico (27°52′N, 110°55′W). This cove has an area of ~11 ha and a mean depth of 3 m, it receives ~80% of the total sewage from the city of Guaymas (~120,000 inhab.), and receives a primary treatment (Figure 1). La Salada Cove has a seasonal pattern of water temperature with maximum of 32°C in August and minimum of 15°C in January; salinity varies from 35 to 37 [19]. The tide is mixed-semidiurnal with a range of 1 m [19]. This region has a dry desert climate with evaporation of ~3000 mm yr^−1^ which exceeds rainfall of <300 mm yr^−1^ [20]. The seasonal pattern of winds is southeastern in summer at 5 m s^−1^ and northwestern in winter at 8–12 m s^−1^, and this pattern also influences coastal circulation [21].

An important consideration is that during the study period, in summer, there was an extreme event of rain due to Tropical Storm Jimena in September 3, 2009. This event collapsed drainage system of the city and urban wastewater was discharged directly to Guaymas Bay; hence, La Salada Cove did not receive wastewater in the summer season and it served as a control period.

### 2.2. Sampling and Measurements

Nutrients and organic matter loading was estimated by its concentrations observed in the discharge site, and the wastewater flow (18600 m^3^ d^−1^) to La Salada Cove. In the discharge site, integrated water samples were collected in each season for the determination of nitrite, nitrate, ammonium, orthophosphate, biochemical oxygen demand, and chemical oxygen demand. Its concentrations were determined by chemical methods [22].

In La Salada Cove, water quality was sampled at 12 sampling stations each week three times in a representative month of each season: winter (February), spring (April), summer (September), and fall (November). Water samples were taken between 7:00 and 13:00 h. At each sampling station, water was collected both near the surface and near the bottom in 1 L plastic bottles; these samples were used to measure nutrients (nitrite, nitrate, ammonium, and orthophosphate) and chlorophyll *a*. Measurements of temperature, salinity, and dissolved oxygen were made at each station using a Hydrolab DS5X multisensor, Hach, Loveland, CO, USA. Water samples were transported on ice to the laboratory for analysis. Nutrient concentration was determined by chemical methods [22]. Samples for analysis of chlorophyll a were collected by filtration through Whatman GF/C glass fiber filters, extracted with 90% *v*/*v* acetone, and measured by spectrophotometry according to [23].

### 2.3. Indexes and Statistical Data Analysis

A multivariate, multidimensional scaling on nonparametric transformed data (log⁡_*x*+1_⁡) was applied and standardized to determine whether seasons had an effect in terms of water quality, the statistical software PRIMER 6 (Primer-E, Ivybridge, UK) was used to perform the analysis.

The Trophic Index (TRIX) based on the pooled effect of oxygen saturation, nitrite, nitrate, ammonium, orthophosphate, and chlorophyll *a* was used to assess water body trophic state according to [10]. The index is given by

(1) $$\begin{array}{c} \text{TRIX}=\frac{\left[\log10\left(\text{Chl}\;a\cdot \text{D}\%\text{O}\cdot \text{N}\cdot \text{P}\right)+1.5\right]}{1.2}, \end{array}$$

where Chl *a* is chlorophyll *a* (*μ*g L-1), D%O is oxygen as an absolute deviation (%) from saturation, N is dissolved inorganic nitrogen N – NO_3_ + NO_2_ + NH_4_ (*μ*M), and P is the total phosphorus P-PO_4_ (*μ*M). TRIX was scaled from 0 to 10, covering a range of four trophic states (0–4 high quality and low trophic level; 4-5 good quality and moderate trophic level; 5-6 moderate quality and high trophic level; 6–10 degraded and very high trophic level).

Also, the Arid Zone Coastal Water Quality Index (AZCI) which is based on the combined effects of dissolved oxygen, nitrite, nitrate, ammonium, and orthophosphate was used to assess water quality [11]. The index is given by

(2) $$\begin{array}{c} \text{AZCI}=\frac{\sum_{i=1}^{n}I_{i}\zeta_{i}}{\sum_{i=1}^{n}\zeta_{i}}, \end{array}$$

where *I*_*i*_ *ζ*_*i*_ is the specific environmental index for variable *i*, *n* is the number of variables, and the range of AZCI is from 0 to 1, where a threshold level of 0.1172 (*≈*0.12) is used to define the overall environmental quality index. Above this level is considered to be of good environmental quality while below this level is of poor quality.

The TRIX and AZCI data grouped by transect for each season were analyzed by comparison of means with one-way ANOVA analysis of variance and box and whisker plot results were obtained. For the statistical analysis of the data, Statgraphics Plus 4.1 was used.

## 3. Results

### 3.1. Nutrients and Organic Matter Loads

La Salada Cove receives 1,237 ton N yr^−1^ and 811 ton P yr^−1^ from sewage. In addition, values of BOD and COD were 3422 and 2503 kg d^−1^, respectively (Table 1).

### 3.2. Seasonal Hydrologic Patterns

**Figure 2:**
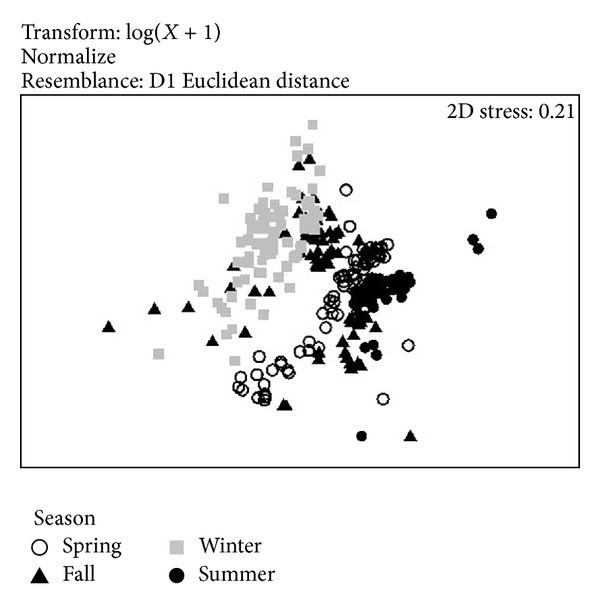
nMDS ordination of water quality parameters for the study area. Temperature, dissolved oxygen, nitrite, nitrate, ammonium, ortophosphate, and chlorophyll *a*, were included in the multivariate analysis.

The hydrological behavior data based on temperature, salinity, dissolved oxygen, nitrite, nitrate, ammonia, orthophosphate, and chlorophyll *a* showed that between winter and summer environmental conditions were significantly different, while in spring and autumn, there were particular hydrological conditions but with greater similarity to each other (Figure 2). The values of these variables are shown in Table 2.

### 3.3. Environmental Indexes

**Figure 3:**
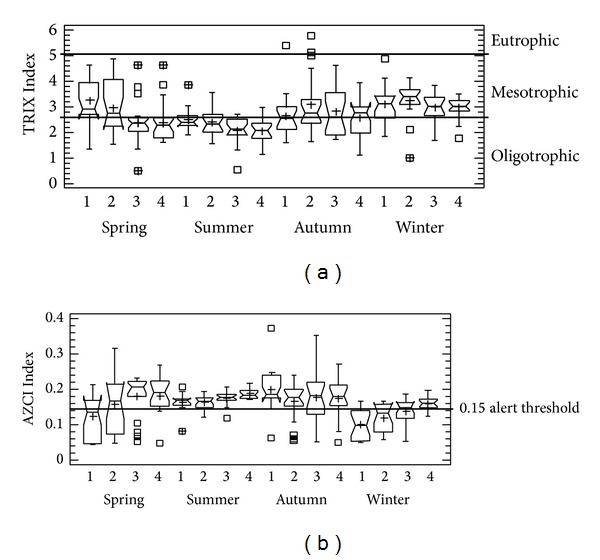
Box and whisker plot of the (a) TRIX, and (b) AZCI for La Salada Cove during seasons. Numbers 1 to 4 represent the transects. Median, quartiles, ranges, and outliers of data are shown for each event. It shows levels of trophic state and water quality influences from urban sewage inputs.

TRIX results indicated that the summer prevailing state was oligotrophic, while in autumn and winter the state mesotrophic was observed. In spring transects 1 and 2 closer to the discharge, a mesotrophic state was found, while in transects 3 and 4 farther away from the discharge, water quality was oligotrophic (Figure 3(a)).

AZCI results indicated better environmental quality in the summer and worse conditions in the autumn and winter. A loss of environmental quality, that is, poor water quality, was observed in spring transects 1 and 2 closer to the discharge, while transects 3 and 4 farther from the discharge had better water quality (Figure 3(b)).

## 4. Discussion

The La Salada Cove urban wastewater receiving primary treatment is insufficient to reduce inputs of N and P. The evidence of the results in this study suggests that these nutrient inputs are associated with increasing trophic state in the coastal ecosystem and where AZCI and TRIX indexes were consistent in indicating symptoms of eutrophication.

Multivariate analysis (nMDS) showed particular hydrological conditions in each season. The marked differences between summer and winter are attributed on the one hand to the water temperature and phytoplankton biomass and on the other hand to La Salada Cove summer conditions where it did not receive nutrient inputs from sewage, while in winter it received input nutrients from sewage and specifically phosphorus concentrations on this season were higher. Also, the water temperature was different by ~12°C between summer and winter and this is a feature typically observed in this region of the Gulf of California. The temperature differences is attributed to the influence of the air temperature in this arid region that has annual oscillations >14°C [17], as well as water bodies of the Gulf region characterized by values of surface temperature of 26°C in summer and 17°C in winter [24].

The effects of urban wastewater discharges to La Salada Cove were observed in two key variables, in addition to being indicated by the TRIX and AZCI. The responses of the body of water to sewage inputs were more evident by the fact that the summer condition in this study served as the reference. In relation to key variables, (1) it was observed in summer that the average chlorophyll *a* concentration was ~6 mg m^−3^, but the maximum values occurred with blooms that were observed exclusively in the other seasons which received loads by sewage, and (2) dissolved oxygen concentrations in summer had average values of ~5 mg L^−1^ and ~5.5 mg L^−1^ in winter, which is substantially lower from that observed in winter (~8 mg L^−1^) in this area of the Gulf of California without sewage influence [24]. The lower values of dissolved oxygen concentration are attributed to the organic load contributed by sewage and indicated by values of BOD and COD. Dissolved oxygen concentrations of water ≥4 mg L^−1^ observed throughout the year both in the medians and the means suggest that the system has sufficient capacity to assimilate the organic load received but does not imply the absence of adverse effects. Concentrations of dissolved oxygen <3 mg L^−1^ were observed in autumn and winter indicating hypoxic events and this represents an impact to the environment and aquatic life.

The TRIX indicated that in summer, when there were no sewage inputs, the system was oligotrophic, while in the other seasons mesotrophic conditions were observed, and this is attributed to nutrient inputs from sewage increasing the rate of supply organic material causing eutrophication symptoms. The TRIX, in general, has been well accepted for application in environmental management actions [25], even established in the Italian environmental legislation [26]; the results of this study suggest that the TRIX can be applicable for monitoring and trophic state evaluation in coastal water bodies of the Gulf of California. In this study, it was notable that, in autumn and winter, the spatial influence of wastewater discharges covered all the system, but in spring the influence was in the first two sampling transects nearest to discharges, which had significant differences to transects that are more distant. This variability is associated with the load of nutrients and organic matter and coastal hydrodynamic conditions prevailing in each season [19–21], considering that La Salada Cove is an open system influenced by seasonal wind patterns that occurs in the Gulf and induces waves [21], coastal circulation as well as tidal mixing [19] which are processes that favor the dilution and assimilation of nutrients and organic matter and generally contribute to this body of water which is less susceptible to eutrophication. This is consistent with observations [27, 28] in the sense that the magnitude of the adverse effects of sewage sources and depends on the magnitude of the discharges of nitrogen and phosphorus, as well as potential dilution and assimilative capacity of each coastal water body.

The AZCI indicated the same pattern as the TRIX on the effects of wastewater discharges in La Salada Cove, showing a poor environmental quality as the median of the data sampling transects is below the alert threshold (0.15) in seasons that the body of water sewage inputs was received, and it was also notable that the first two transects closest to the discharge area had the worst environmental quality. Previous studies showed that AZCI also worked to characterize seasonal changes by environmental effects (rain and coastal processes) [11] and anthropogenic influences [18] in coastal lagoons from the Gulf of California.

AZCI and TRIX indices differ primarily in that (1) the first incorporates chlorophyll *a*, a biological variable expressing phytoplankton biomass, and (2) the second used algebraic function involving different scale and interpretation, in addition to the fact that AZCI applicates standardization process of the inverses values of the variables and the lowest concentration is the weight value which is more important. In particular, the average chlorophyll *a* value was <2.5 relatively low for seasons, where there were sewage inputs, but eventually had extreme values that might make a difference between TRIX and AZCI. In practical terms, the two water indices provided similar results and were consistent with the environmental condition of La Salada Cove. Taking this into consideration, priority should be given to the development of reliable indicators of trophic levels of coastal ecosystems; also it would be useful to identify policies aimed at establishing environmental legislation in order to help abate the causes of the process of eutrophication [29].

This study provides evidence of the process of eutrophication in the coastal ecosystem of La Salada Cove; this information is useful since it is necessary to increase the amount of data of many coastal water bodies in Mexico [30], in order to compare coastal scenarios in other parts of the world in order to achieve a better understanding of the eutrophication processes [31]. The challenge is to establish a strategy to reduce nutrient inputs from urban wastewater in order to maintain and prevent the loss of ecosystem services provided by coastal ecosystems. In particular, to explore cases where the cost benefit of control measures of excessive nutrient loading is considered and evaluated, there are limited case studies; a good example is the recent work done in the Gulf of Finland [32].

## Figures and Tables

**Table 1:** Inputs of organic matter and inorganic dissolved nutrients of La Salada Cove.

Variable	Load kg d^−1^
Biochemical oxygen demand (5 days)	3422
Chemical oxygen demand	12503
Soluble phosphorus	307
Nitrite	<1
Nitrate	4
Ammonium	375
Total dissolved inorganic nitrogen	417

**Table 2:** Values of temperature, salinity, dissolved oxygen, nitrite, nitrate, ammonium, orthophosphate, and chlorophyll *a* around year in La Salada Cove.

	Spring			Summer			Fall			Winter		
	Mean ± SD	Median	Min. − Max.	Mean ± SD	Median	Min. − Max.	Mean ± SD	Median	Min. − Max.	Mean ± SD	Median	Min. − Max.
Temperature (°C)	27.4 ± 0.5	28.1	27.2 − 29.7	29.3 ± 0.8	29.6	28.0 − 30.2	22.4 ± 2.8	21.2	17.9 − 26.9	16.9 ± 1.1	16.9	14.4 − 18.9
DO (mg L^−1^)	4.3 ± 0.7	4.2	3.4 − 6.7	5.6 ± 0.3	5.6	4.7 − 6.1	5.8 ± 1.7	5.6	2.5 − 8	5.5 ± 1.6	5.8	1.7 − 8.1
N-NO_2_^−^ (*μ*M)	0.218 ± 0.47	0.131	0 − 3.87	1.798 ± 1.63	1.277	0.35 − 9.11	0.511 ± 0.38	0.378	0.03 − 1.46	0.661 ± 0.37	0.521	0.01 − 1.32
N-NO_3_ (*μ*M)	0.145 ± 0.17	0.074	0.01 − 0.96	0.09 ± 0.08	0.064	0.02 − 0.46	0.359 ± 0.25	0.377	0.03 − 0.82	0.517 ± 0.23	0.456	0.06 − 0.94
N-NH_4_ (*μ*M)	5.82 ± 6.83	19.79	0.23 − 19.31	1.733 ± 2.6	0.806	0.05 − 16.22	4.371 ± 6.13	1.197	0.01 − 19.09	1.609 ± 1.21	1.238	0.09 − 3.99
PO_4_ (*μ*M)	4.291 ± 6.38	1.231	0.34 − 21.98	1.495 ± 0.55	1.26	1.07 − 5.1	2.439 ± 3.4	13.54	0.32 − 16.42	5.014 ± 3.03	2.184	1.38 − 21.98
Chl a (mg m^−3^)	0.9 ± 1.8	0.4	0 − 13.7	5.7 ± 0.7	5.6	3.4 − 6.7	2.5 ± 5.6	0.8	0 − 37.2	2.2 ± 2.9	1	0 − 16.9
